# Peritoneum as the sole distant metastatic site of lung adenosquamous cell carcinoma: a case report

**DOI:** 10.1186/s13256-017-1431-z

**Published:** 2017-09-27

**Authors:** Pan Yang, Wei-Liang Li, Jeff-X Zhou, Yu-Bo Yang, Xia-Xiang Jin

**Affiliations:** 1 Department of Pathology, Ningbo University School of Medicine, Ningbo, China; 2 Department of Respiratory Medicine, Mingzhou Hospital, Zhejiang University, No. 168 West Taian Road, Ningbo, 315199 China; 3 Department of Respiratory Medicine, 113th Hospital of PLA, No. 377 East Zhongshan Road, Ningbo, 315040 China; 4 Department of Pathology, 113th Hospital of PLA, No. 377 East Zhongshan Road, Ningbo, 315040 China

**Keywords:** Lung adenosquamous cell carcinoma, Peritoneum, Metastasis, *KRAS* mutation

## Abstract

**Background:**

Peritoneum metastasis of lung cancer is a rare event which, in addition to the peritoneum, usually involves multiple metastatic tissues. Here we report a case of a patient with lung adenosquamous cell carcinoma with the peritoneum as the sole distant metastatic site.

**Case presentation:**

An 82-year-old Han Chinese man, in the teaching profession, was diagnosed with lung adenosquamous cell carcinoma in the upper lobe of his left lung with the involvement of ipsilateral hilar and mediastinal lymph nodes, and was initially staged as IIIa (cT_2_N_2_M_0_). Molecular testing identified a mutation at *KRAS* G12A. Due to his poor physical condition, our patient was given gamma knife radiotherapy with a total dose of 28.0 Gy. Two weeks later, our patient was diagnosed as peritoneal metastasis identified by using magnetic resonance imaging and confirmed with ascitic cytology and peritoneal histology. No other distant metastatic sites such as liver, brain, bone, paranephroi, and lungs were found. Subsequently, our patient received palliative intraperitoneal chemotherapy, and died within 2 months.

**Conclusions:**

Our patient represented a rare case of lung adenosquamous cell carcinoma harboring the *KRAS* G12A mutation, which metastasized distantly to the peritoneum only, and progressed rapidly.

## Background

Metastasis is the predominant cause of cancer-related death. For lung cancer, 40–60% patients have metastasis at diagnosis [1]. The most common metastatic sites for lung cancer include the brain, bone, and liver, among others [2]. The incidence of peritoneal metastasis of lung cancer is less than 5% [3, 4]. For lung cancer patients with peritoneal metastases, the prognosis is poor with a overall survival time of usually less than 2 months [3–10]. Kirsten rat sarcoma (*KRAS*) gene mutation is one of the key drivers for lung cancer development [11, 12], and is detected in 6–30% of non-small-cell lung cancer (NSCLC) patients [11, 13–16]. Patients with *KRAS* mutations can easily develop metastasis, and usually have poor prognosis [12, 17–19]. In this study, we report an uncommon case of a patient with lung adenosquamous cell carcinoma harboring *KRAS* G12A mutation with the peritoneum as the sole distant metastatic site.

## Case presentation

**Fig. 1:**
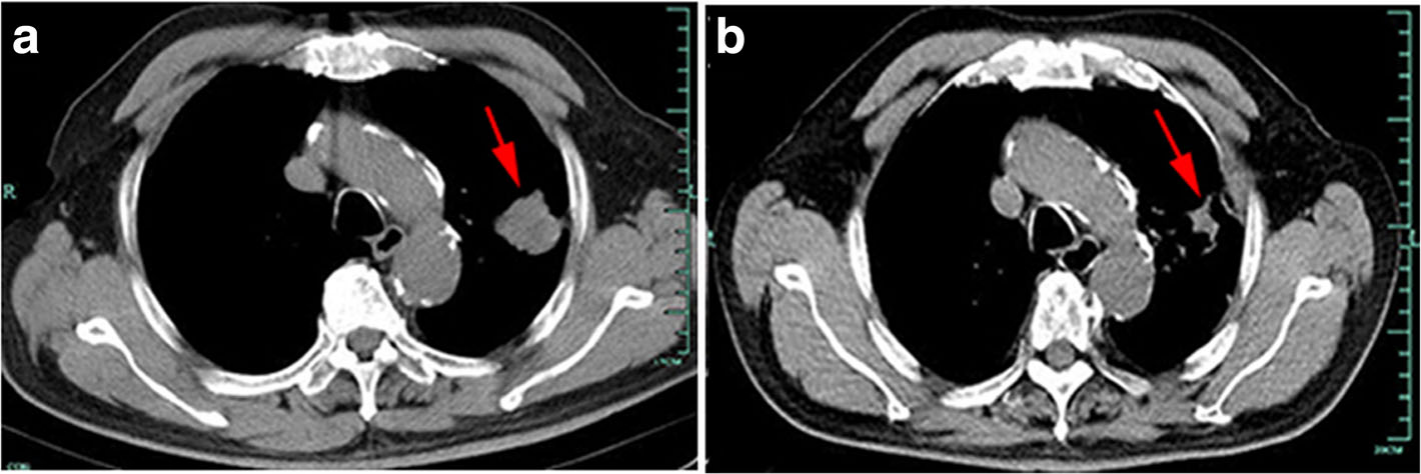
**a** Chest computed tomography scan showed a mass in the upper lobe of the left lung (*red arrow*).** b** A chest computed tomography scan revealed that there was a reduction in the size of the mass in the upper lobe of the left lung after radiotherapy (*red arrow*)

**Fig. 2:**
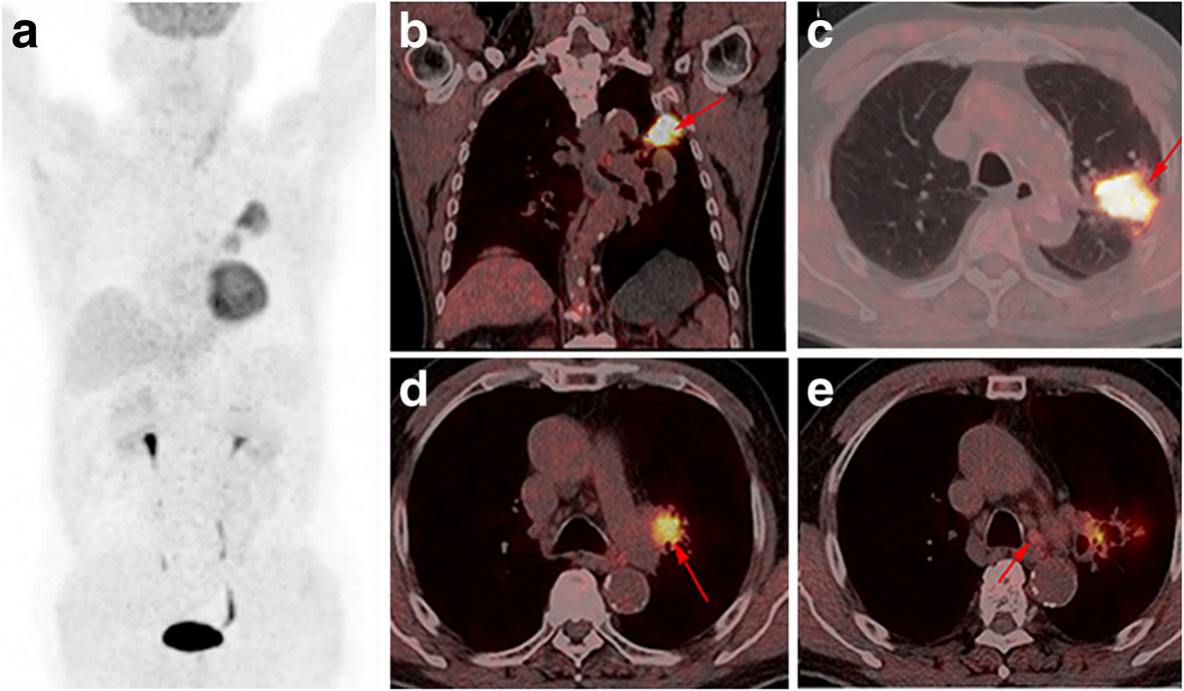
**a** A positron emission tomography-computed tomography scan shows two hypermetabolic lesions in the upper lobe of the left lung and ipsilateral hilar lymph node. **b**-**c** Axial view of positron emission tomography-computed tomography scans for hypermetabolic lesion in the upper lobe of his left lung (*red arrows*), **d** in the left hilar lymph node (*red arrow*) and **e** in the left mediastinal lymph node (*red arrow*)

**Fig. 3:**
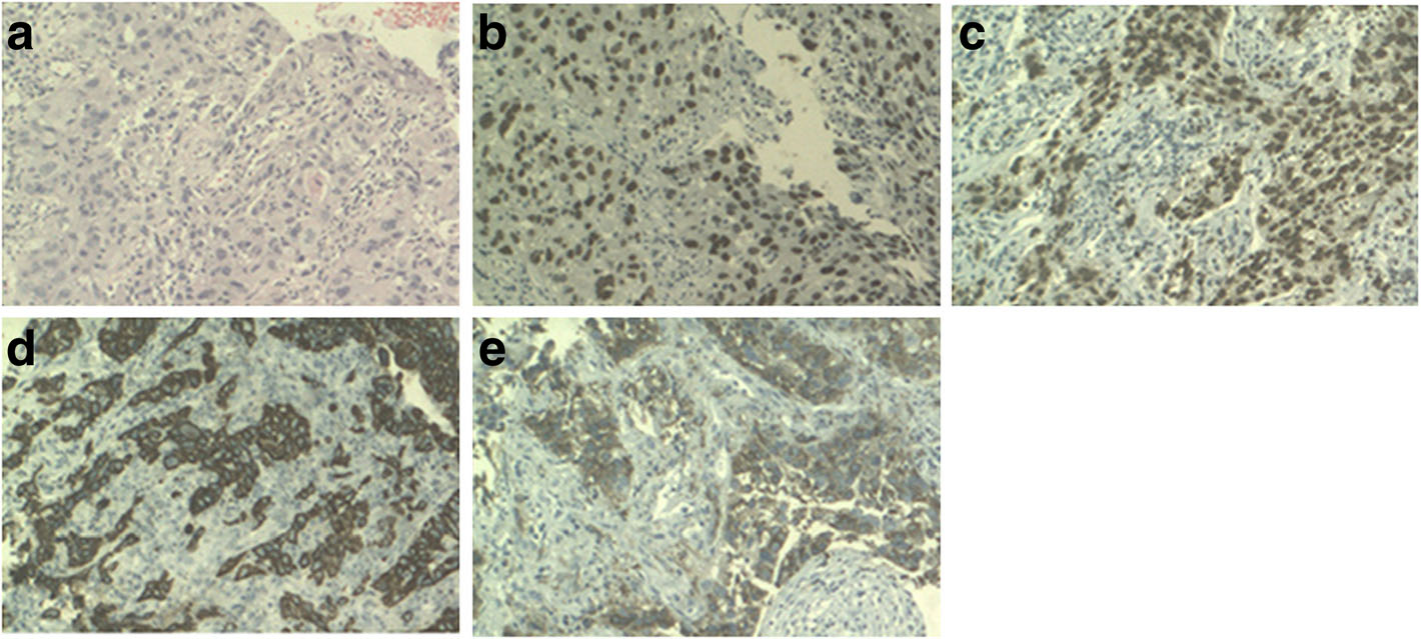
**a** The tumor specimen shows adenosquamous cell carcinoma with moderate differentiation (hematoxylin and eosin staining, magnification × 40). **b** Immunohistochemical of the tumor specimen is positive for p63 (positive in nucleus, magnification × 40), **c** thyroid transcription factor-1 (positive in nucleus, magnification × 40), **d** cytokeratin 7 (positive in cytoplasm, magnification × 40), **e** napsin-A (positive in cytoplasm, magnification × 40)

**Fig. 4:**
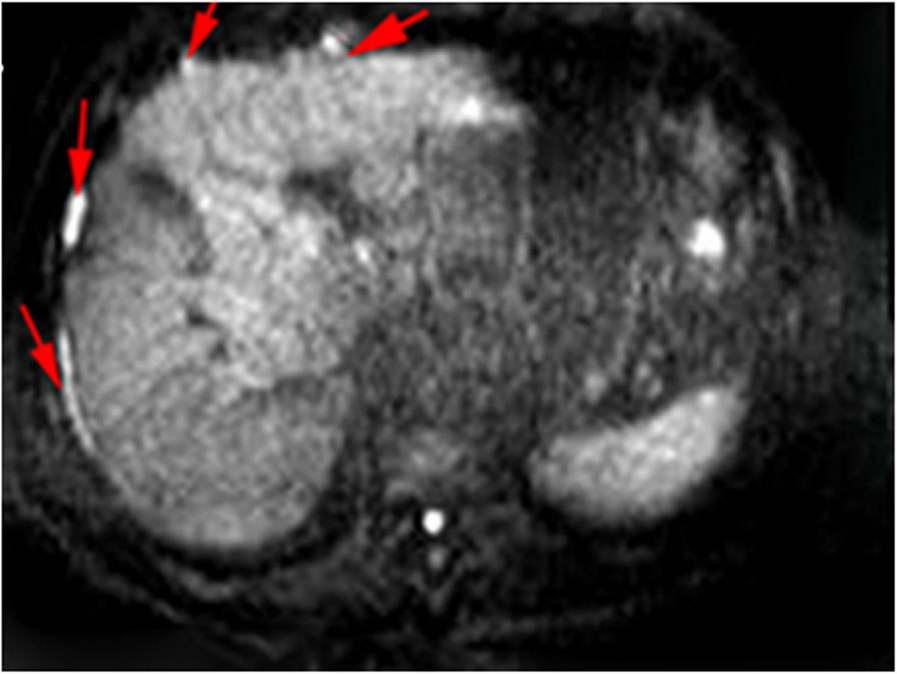
Abdominal enhanced magnetic resonance image analysis showed multiple nodule-like lesions in the hepatic capsule (*red arrows*)

**Fig. 5:**
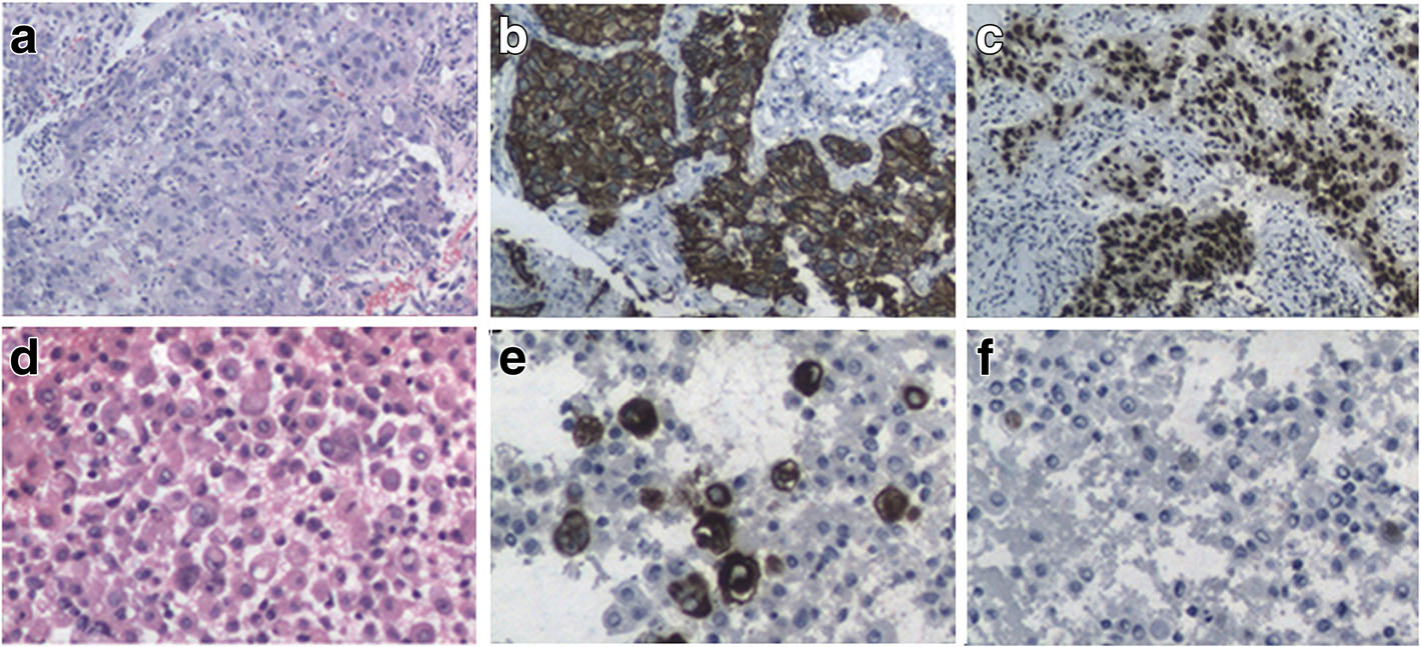
**a** Cytological examination of peritoneum tissue (hematoxylin and eosin staining, magnification × 40). **b** Immunohistochemical of peritoneum tissue showed positive for cytokeratin 7 (magnification × 40) and **c** thyroid transcription factor-1 (magnification × 40). **d** Cytological examination of ascites tumor cells (hematoxylin and eosin staining, magnification × 100). **e** Immunohistochemistry of ascite tumor cells showed positive for cytokeratin 7 (magnification × 100) and **f** thyroid transcription factor-1 (magnification × 100)

An 82-year-old Han Chinese man, in the teaching profession, was admitted to our hospital for cough and bloody sputum. Our patient did not have fever, chest pain, dizziness, headache, nausea, or vomiting. He had been an ex-smoker for 30 years, had had hypertension for more than 60 years, and had a history of coronary heart disease with myocardial infarction 10 years earlier. A physical examination showed that his vital signs were stable without edema, cyanosis, or lymphadenopathy. Blood test results showed increases in the counts of white blood cells (WBC) and neutrophils. Serum levels of cytokeratin (CK)-19 fragment CYFRA21-1 and CA153 were 3.57 ng/mL and 47.2U/mL, respectively. A chest computed tomography (CT) scan revealed a mass measuring 30 × 30 mm in size in the upper lobe of his left lung with an enlarged left hilum (Fig. 1a). A positron emission tomography-computed tomography (PET-CT) scan showed three hypermetabolic lesions in the upper lobe of his left lung, left hilum, and left mediastinal lymph node (Fig. 2). No distant metastatic sites such as liver, brain, bone, paranephroi, lungs, and peritoneum were found. Bronchoscopy revealed an external compression of the upper lobe of his left lung. A CT-guided percutaneous lung biopsy was carried out, and the histological examination showed adenosquamous cell carcinoma. Immunohistochemical (IHC) studies showed positive outcomes in thyroid transcription factor-1 (TTF-1), napsin-A (NAP-A), CK-7, and P63, and negative outcome in CK-20 (Fig. 3). Deoxyribonucleic acid (DNA) sequencing showed that the tumor harbored *KRAS* G12A mutation. Based on the clinical findings, our patient was staged as IIIa (T_2_N_2_M_0_). Because of his poor physical condition, our patient was given palliative gamma knife radiotherapy with a total dose of 28.0 Gy for one cycle combined with anti-infective therapy. The WBC count returned to the normal range and his vital signs were stable following treatment, and our patient was discharged at his request after hospitalization for 43 days. Ten days after discharge, he was admitted to the hospital again because of weakness and anorexia. Blood test results showed increases in WBC and neutrophil counts. Serum levels of CA125, CA199, and CA153 were 474.3U/mL, 467.5 U/mL, and 28.4 U/mL respectively. A chest CT scan revealed that there was a reduction in the size of the original tumor in the upper lobe of his left lung (Fig. 1b). Abdominal enhanced magnetic resonance image (MRI) analysis showed multiple nodule-like lesions in the hepatic capsule, and was considered as metastatic carcinoma in the peritoneum (Fig. 4). Peritoneal effusion and peritoneum biopsy were evidenced, and a cytological examination of the ascites found clustered and scattered tumor cells; IHC of peritoneum tissue and ascite tumor cells showed positive for CK-7 and TTF-1 (Fig. 5). No other distant metastasis was identified. Based on the progression of the disease, our patient was diagnosed with stage IV lung cancer with distant metastasis to the peritoneum (cT_2_N_2_M_1_). Accordingly, our patient received palliative intraperitoneal chemotherapy (cisplatin 40 mg combined with recombinant human endostatin 45 mg) for one time. Unfortunately, no improvements were achieved and our patient died 11 days after readmission (Table 1).

**Table 1:** Timeline of the patient

Dates	Course	Events
About Mar 28, 2016	At least day -10	Onset
Apr 7, 2016	Day 1	First hospitalization
Apr 12, 2016	Day 6	Bronchoscopy
Apr 15, 2016	Day 9	PET-CT
Apr 25, 2016	Day 19	Lung biopsy
May 6, 2016	Day 30	Gamma knife radiotherapy (started)
May 19, 2016	Day 43	Gamma knife radiotherapy (ended)
May 20, 2016	Day 44	*KRAS* G12A mutation reported
May 20, 2016	Day 44	Hospital discharge
May 30, 2016	Day 54	Readmission
May 31, 2016	Day 55	Peritoneocentesis
Jun 4, 2016	Day 59	MRI
Jun 6, 2016	Day 61	Peritoneal biopsy
Jun 8, 2016	Day 63	Intraperitoneal chemotherapy
Jun 10, 2016	Day 65	Died

*PET-CT* positron emission tomography-computed tomography, *KRAS* Kirsten rat sarcoma, *MRI* magnetic resonance imaging

## Discussion

Peritoneum is a common metastatic site for gastric cancer [20], colorectal cancer [21], and ovarian cancer [22], but uncommon for lung cancer with its incidence rate about 1–5% [3, 4]. In the reported cases involving peritoneal metastasis of lung cancer, there are usually multiple other distant metastatic tissues including brain, liver, and so on [4]. In the present case, it is reported as a lung adenosquamous cell carcinoma with peritoneal metastasis as the sole distant metastatic site, which was different from what was reported previously in which the pathological type included adenocarcinoma, squamous cell carcinoma, and large cell carcinoma [3, 4].

The patient in the present case had rapid progression in his clinical course. There may be several reasons underlying the rapid progression of this disease. First of all, his general condition, including his age, hypertension, infection, and so on, had limited the treatment options. As a result, only palliative radiotherapy was administrated. Second, the pathological type of adenosquamous cell carcinoma may also have contributed to the rapid worsening of our patient’s condition. It has been reported that adenosquamous cell carcinoma of the lung possesses strong invasion ability and usually metastasizes at an early stage [23–25], and peritoneal metastasis is usually followed by particularly poor prognosis [3, 4, 7]. The third, genetic alterations such as *KRAS* mutations can increase the activities of matrix metalloproteases, cysteine proteases, serine proteases, urokinase plasminogen activator, and enzymes responsible for tumor invasion and metastasis [26–28]. In the present case, the mutation in *KRAS* may have played a pivotal role in the metastasis of lung cancer. Li *et al*. reported a case of lung squamous cell carcinoma with *BRAF* V600E and *KRAS* G12A mutations with peritoneal metastasis [29]. In addition, Pal S et al. reported that lung cancer with *KRAS* mutation had an increased incidence of distant metastasis [30].

## Conclusions

In summary, according to the present case and reported literature, lung adenosquamous cell carcinoma with *KRAS* mutations has high susceptibility to distant metastasis including peritoneal metastasis and a very rapid progression of clinical course. Sometimes the peritoneum may be the sole distant metastatic site and be followed by particularly poor prognosis.

## Abbreviations

CA: Carbohydrate antigen
CK: Cytokeratin
CT: Computed tomography
CYFRA21-1: Cytokeratin 19 fragment
DNA: Deoxyribonucleic acid
Gy: Gray
IHC: Immunohistochemical
KRAS: Kirsten rat sarcoma
MRI: Magnetic resonance image
NAP-A: Napsin-A
NSCLC: Non-small-cell lung cancer
PET-CT: Positron emission tomography-computed tomography
TTF-1: Thyroid transcription factor-1
WBC: White blood cells

## References

[1] Jemal A, Siegel R, Xu J, *et al.* Cancer statistics, 2010. CA Cancer J Clin. 2010;60:277–300. 10.3322/caac.20073 20610543

[2] Quint LE, Tummala S, Brisson LJ, *et al.* Distribution of distant metastases from newly diagnosed non-small cell lung cancer. Ann Thorac Surg. 1996;62:246–50. 10.1016/0003-4975(96)00220-2 8678651

[3] Su HT, Tsai CM, Perng RP. Peritoneal carcinomatosis in lung cancer. Respirology. 2008;13:465–7. 10.1111/j.1440-1843.2008.01268.x 18399874

[4] Satoh H, Ishikawa H, Yamashita YT, *et al.* Peritoneal carcinomatosis in lung cancer patients. Oncol Rep. 2001;8:1305–7. 10.3892/or.8.6.1305 11605054

[5] Bazine A, Fetohi M, Khmamouch MR, *et al.* An unusual case of isolated peritoneal metastases from lung adenocarcinoma. Case Rep Oncol. 2014;7:600–4. 10.1159/000366293 PMC4178321 25298766

[6] Tanriverdi O, Barutca S, Meydan N. Relapse with isolated peritoneal metastasis in lung adenocarcinoma: case report and review of the literature. Contemp Oncol (Pozn). 2012;16:586–9. 10.5114/wo.2012.32495 PMC3687476 23788949

[7] Hanane K, Salma B, Khadija B, *et al.* Peritoneal carcinomatosis, an unusual and only site of metastasis from lung adenocarcinoma. Pan Afr Med J. 2016;23:60. 10.11604/pamj.2016.23.60.8910 PMC4862789 27217885

[8] Sereno M, Rodriguez-Esteban I, Gomez-Raposo C, *et al.* Lung cancer and peritoneal carcinomatosis. Oncol Lett. 2013;6:705–8. 10.3892/ol.2013.1468 PMC3789112 24137394

[9] Song SH, Oh YJ, Kim YN, *et al.* Squamous cell carcinoma of the lung with simultaneous metastases to peritoneum and skeletal muscle. Thorac Cancer. 2014;5:101–3. 10.1111/1759-7714.12034 PMC4704279 26766983

[10] Du C, Li Z, Wang Z, *et al.* Stereotactic aspiration combined with gamma knife radiosurgery for the treatment of cystic brainstem metastasis originating from lung adenosquamous carcinoma: A case report. Oncol Lett. 2015;9:1607–13. 10.3892/ol.2015.2968 PMC4356421 25789009

[11] Yu HA, Sima CS, Shen R, *et al.* Prognostic impact of KRAS mutation subtypes in 677 patients with metastatic lung adenocarcinomas. J Thorac Oncol. 2015;10:431–7. 10.1097/JTO.0000000000000432 PMC4479120 25415430

[12] Martin P, Leighl NB, Tsao MS, *et al.* KRAS mutations as prognostic and predictive markers in non-small cell lung cancer. J Thorac Oncol. 2013;8:530–42. 10.1097/JTO.0b013e318283d958 23524403

[13] Roberts PJ, Stinchcombe TE, Der CJ, *et al.* Personalized medicine in non-small-cell lung cancer: is KRAS a useful marker in selecting patients for epidermal growth factor receptor-targeted therapy? J Clin Oncol. 2010;28:4769–77. 10.1200/JCO.2009.27.4365 20921461

[14] Zheng D, Wang R, Zhang Y, *et al.* The prevalence and prognostic significance of KRAS mutation subtypes in lung adenocarcinomas from Chinese populations. Onco Targets Ther. 2016;9:833–43. 10.2147/OTT.S96834 PMC4768896 26955281

[15] Riely GJ, Kris MG, Rosenbaum D, *et al.* Frequency and distinctive spectrum of KRAS mutations in never smokers with lung adenocarcinoma. Clin Cancer Res. 2008;14:5731–4. 10.1158/1078-0432.CCR-08-0646 PMC2754127 18794081

[16] Gou LY, Niu FY, Wu YL, *et al.* Differences in driver genes between smoking-related and non-smoking-related lung cancer in the Chinese population. Cancer. 2015;121 Suppl 17:3069–79. 10.1002/cncr.29531 26331813

[17] Massarelli E, Varella-Garcia M, Tang X, *et al.* KRAS mutation is an important predictor of resistance to therapy with epidermal growth factor receptor tyrosine kinase inhibitors in non-small-cell lung cancer. Clin Cancer Res. 2007;13:2890–6. 10.1158/1078-0432.CCR-06-3043 17504988

[18] Ricciuti B, Leonardi GC, Metro G, *et al.* Targeting the KRAS variant for treatment of non-small cell lung cancer: potential therapeutic applications. Expert Rev Respir Med. 2016;10:53–68. 10.1586/17476348.2016.1115349 26714748

[19] Marabese M, Ganzinelli M, Garassino MC, *et al.* KRAS mutations affect prognosis of non-small-cell lung cancer patients treated with first-line platinum containing chemotherapy. Oncotarget. 2015;6:34014–22. 10.18632/oncotarget.5607 PMC4741822 26416458

[20] Riihimaki M, Hemminki A, Sundquist K, *et al.* Metastatic spread in patients with gastric cancer. Oncotarget. 2016;7:52307–16. 10.18632/oncotarget.10740 PMC5239553 27447571

[21] Riihimaki M, Hemminki A, Sundquist J, *et al.* Patterns of metastasis in colon and rectal cancer. Sci Rep. 2016;6:29765. 10.1038/srep29765 PMC4945942 27416752

[22] Pereira A, Perez-Medina T, Magrina JF, *et al.* International Federation of gynecology and obstetrics staging classification for cancer of the ovary, fallopian tube, and peritoneum: estimation of survival in patients with node-positive epithelial ovarian cancer. Int J Gynecol Cancer. 2015;25:49–54. 10.1097/IGC.0000000000000316 25405578

[23] Sridhar KS, Bounassi MJ, Raub Jr W, *et al.* Clinical features of adenosquamous lung carcinoma in 127 patients. Am Rev Respir Dis. 1990;142:19–23. 10.1164/ajrccm/142.1.19 2368969

[24] Shimizu J, Oda M, Hayashi Y, *et al.* A clinicopathologic study of resected cases of adenosquamous carcinoma of the lung. Chest. 1996;109:989–94. 10.1378/chest.109.4.989 8635382

[25] Maeda H, Matsumura A, Kawabata T, *et al.* Adenosquamous carcinoma of the lung: surgical results as compared with squamous cell and adenocarcinoma cases. Eur J Cardiothorac Surg. 2012;41:357–61. 10.1016/j.ejcts.2011.05.050 21737295

[26] Jankun J, Maher VM, McCormick JJ. Malignant transformation of human fibroblasts correlates with increased activity of receptor-bound plasminogen activator. Cancer Res. 1991;51:1221–6. 1847659

[27] Buo L, Meling GI, Karlsrud TS, *et al.* Antigen levels of urokinase plasminogen activator and its receptor at the tumor-host interface of colorectal adenocarcinomas are related to tumor aggressiveness. Hum Pathol. 1995;26:1133–8. 10.1016/0046-8177(95)90276-7 7557947

[28] Yamamoto H, Itoh F, Senota A, *et al.* Expression of matrix metalloproteinase matrilysin (MMP-7) was induced by activated Ki-ras via AP-1 activation in SW1417 colon cancer cells. J Clin Lab Anal. 1995;9:297–301. 10.1002/jcla.1860090504 8531010

[29] Li B, Lu JC, He D, *et al.* Rapid onset lung squamous cell carcinoma with prominent peritoneal carcinomatosis and an eosinophilic leukemoid reaction, with coexistence of the BRAF V600E and oncogenic KRAS G12A mutations: a case report. Oncol Lett. 2014;8:589–93. 10.3892/ol.2014.2169 PMC4081353 25013473

[30] Pal S, Amin PJ, Sainis KB, *et al.* Potential Role of TRAIL in metastasis of mutant KRAS expressing lung adenocarcinoma. Cancer Microenviron. 2016;9:77–84. 10.1007/s12307-016-0184-3 PMC5264657 27106232

